# Supplementary figures and images for: DSGRec: dual-path selection graph for multimodal recommendation
Source: PeerJ Comput Sci. 2025 Apr 15;11:e2779. doi: 10.7717/peerj-cs.2779 (PMC12190342; doi:10.7717/peerj-cs.2779)

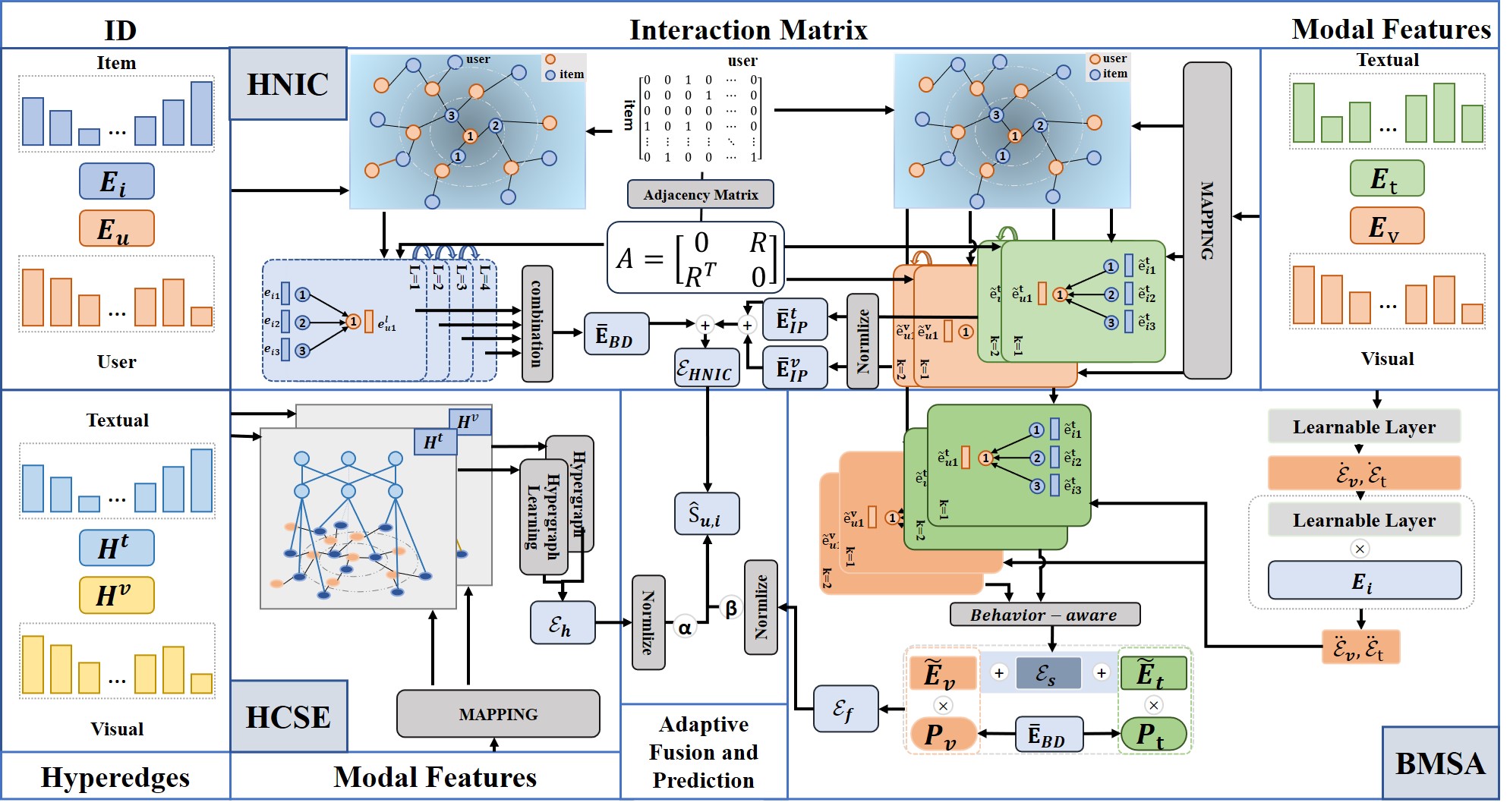

Supplement: Supplemental Information 1 [file peerj-cs-11-2779-s001.zip › DSGRec/img/framework.jpg]
